# Effect of an Online, Interactive Lifestyle Intervention Program on 12-Month Disease Management Outcomes: Protocol for The Healthy Living in Inflammatory Arthritis (HELIA) Randomized Controlled Trial

**DOI:** 10.2196/83749

**Published:** 2026-05-25

**Authors:** Kim van Slingerland, Nathalie Wilmsen, Emma Coles, Radboud J E M Dolhain, Pascal H P de Jong

**Affiliations:** 1 Department of Rheumatology, Erasmus Medical Center, Dr. Molenwaterplein 40, Rotterdam, 3015GD, The Netherlands, 31650032385; 2 Voeding Leeft, Amsterdam, The Netherlands

**Keywords:** lifestyle intervention, rheumatoid arthritis, psoriatic arthritis, disease activity, cost-effectiveness, patient-reported outcomes, disease burden

## Abstract

**Background:**

Innovative disease management strategies have significantly improved clinical outcomes in inflammatory arthritis (IA). Although more than 80% of patients achieve low disease activity (LDA), the disease continues to exert a considerable impact on patients’ lives. For these patients with IA, a lifestyle intervention program may add value by reducing inflammatory activity, potentially alleviating the disease burden and the risk of a flare.

**Objective:**

This study aimed to compare 12-month disease management outcomes between patients with rheumatoid arthritis (RA) and psoriatic arthritis (PsA) with LDA who follow an online, interactive lifestyle intervention program (Leef! met Reuma) and those who receive general nutrition recommendations offered as part of usual care.

**Methods:**

The Healthy Living in Inflammatory Arthritis (HELIA) trial is an open-label, randomized controlled clinical trial with the aim of enrolling 200 adult patients diagnosed with RA or PsA according to the American College of Rheumatology or European Alliance of Associations for Rheumatology 2010 criteria, or Classification for Psoriatic Arthritis criteria, respectively. Other inclusion criteria were (1) having LDA defined as Disease Activity Score ≥2.6 and <3.5 or Disease Activity in Psoriatic Arthritis >4 and <16 and (2) stable disease-modifying antirheumatic drug dosages for ≥6 months prior to randomization. Patients are randomized into a lifestyle intervention or general nutrition recommendations using a 1:1 allocation ratio. The lifestyle intervention program consists of a 6-month intensive part, followed by an 18-month aftercare period. During the intensive part, there were 5 online Zoom (Zoom Communications Inc) meetings and 7 coaching sessions. The program focuses on 4 pillars, namely nutrition, exercise, relaxation, and sleep. The prescribed diet resembles the Mediterranean diet, with an emphasis on unprocessed foods. The general nutrition recommendations are based on the Dutch food-based dietary guidelines. The primary objective is the proportional difference in Disease Activity Score and Disease Activity in Psoriatic Arthritis remission and/or successful tapering of disease-modifying antirheumatic drug treatment after 12 months of follow-up. Meeting either criterion is sufficient to qualify for the primary outcome. Additionally, a cost-effectiveness analysis will be conducted using the incremental cost-effectiveness ratio as the outcome. Other secondary objectives include health risk and clinical and patient-reported outcomes over time and at specific time points. Primary and secondary outcomes are analyzed according to an intention-to-treat principle.

**Results:**

The trial was registered in the ISRCTN (International Standard Randomised Controlled Trial Number) registry retrospectively after recruitment had begun, due to administrative issues. The authors acknowledge this deviation.

**Conclusions:**

A randomized controlled trial is performed to compare the effectiveness of an online, interactive lifestyle intervention program and general nutrition recommendations in patients with IA with LDA from a clinical, patient, and societal point of view. If the intervention is proven effective, it can be applied as a complementary option to current treatment approaches.

## Introduction

Clinical outcomes of patients with an inflammatory arthritis (IA), including rheumatoid arthritis (RA) and psoriatic arthritis (PsA), have improved enormously due to further development of disease management strategies [1]. As a result, >80% of the patients with an IA reach low disease activity (LDA) [1]. Nevertheless, the disease still continues to exert a considerable impact on patients’ lives, manifesting as persistent fatigue, pain, and morning stiffness [2]. This residual disease activity is less in patients with IA who are in remission. Unfortunately, remission occurs less often and is not always achievable with current disease-modifying antirheumatic drug (DMARD) treatment. Irrespective of the residual disease activity, patients with IA frequently express a desire to taper their medication, even with the risk of a disease flare, due to concerns about side effects (ie, infections). For these patients, a lifestyle intervention program might be of added value, as increasing evidence suggests that lifestyle factors interfere with inflammatory activity and, consequently, a lifestyle intervention aimed at reducing inflammatory activity might lessen disease burden and the risk of disease flares [3-6].

Furthermore, previous research showed that patients with IA can undertake steps to improve their dietary quality as a possible strategy to manage arthritis [7]. Other studies also showed that certain diets, such as the Mediterranean diet, may add value for patients with IA [8-12]. Additional lifestyle factors, such as physical activity, sleep, and stress, also influence the course of IA [13-15]. Some researchers even state that lifestyle interventions should be the basis of IA treatment [15].

A recent lifestyle intervention trial, called “Plants for Joints,” in patients with RA and osteoarthritis showed promising outcomes. Patients with RA showed significant improvements in metabolic status and C-reactive protein (CRP) levels (median change −1.2, IQR –2.1 to –0.3). Disease activity also improved, demonstrated by a significantly lower Disease Activity Score (DAS28) after 1 year of the intervention, with sustainment of this improvement in the second year of follow-up (mean DAS28 change from baseline −0.9, SD –1.2 to –0.6). Consequently, half of the patients with RA were able to taper their medication, an effect that also persisted during the entire follow-up [16,17].

In line with this, Voeding Leeft, a nonprofit organization, developed a lifestyle program for patients with a rheumatic disease (Leef! met Reuma). This lifestyle intervention is aimed at achieving a lasting behavioral change (based on the I-Change model) [18]. Voeding Leeft already offers other lifestyle programs, including Reverse Diabetes 2 Now, with proven efficacy [19]. A pilot study that investigated the effect of Leef! met Reuma showed promising results, specifically improvement of health risks such as a reduction in weight, BMI, and waist circumference, and a reduction in disease burden [20]. However, the aforementioned outcomes were based on self-reported data, patients were not randomized, and there were no data on disease activity in patients with IA.

Therefore, the primary objective of this study is to compare 12-month disease management outcomes between patients with RA and PsA with LDA who follow an online, interactive lifestyle intervention program (Leef! met Reuma) and those who receive general nutrition recommendations, by measuring the proportional difference in DAS28 and Disease Activity in Psoriatic Arthritis (DAPSA) remission and/or successful tapering of DMARD treatment. Meeting either criterion is sufficient to qualify for the primary outcome.

Secondary objectives are:

- To evaluate the cost-effectiveness of the online lifestyle intervention program versus general nutrition recommendations, by using the incremental cost-effectiveness ratio (ICER) as the outcome, which is the ratio of the difference in costs to incremental benefits between both interventions.
- To compare health risk (weight, BMI, and waist circumference) between the lifestyle intervention program and general nutrition recommendations.
- To compare disease impact between an online, interactive lifestyle intervention program and general nutrition recommendations offered as part of usual care in patients with IA with LDA by assessing the difference in patient-reported outcomes (PROs), namely general health (GH), pain, morning stiffness, fatigue, functional ability, and quality of life, over time and specific time points.

## Methods

### Trial Design and Setting

The Healthy Living in Inflammatory Arthritis (HELIA) trial is an open-label, randomized controlled trial and takes place at the Erasmus MC, an academic hospital in Rotterdam, The Netherlands.

### Recruitment and Eligibility Criteria

#### Recruitment

Patients with RA or PsA who have reached LDA but still experience disease-related problems are asked by their treating rheumatologist to participate in the study. If they are interested in participating in the trial, they will be given the patient information folder. Once patients have given their consent after a reflection period of ≥48 hours, the research nurse (RN) will check whether the patient meets the inclusion criteria. If trial participation is declined, then patients will be treated according to the insight of the treating rheumatologist using current treatment recommendations as a guideline. By signing the informed consent, patients authorize the retrieval of necessary data from their medical records for the study.

#### Inclusion Criteria

The inclusion criteria for this study are as follows:

1. Diagnosed with RA or PsA according to the American College of Rheumatology or European Alliance of Associations for Rheumatology (EULAR) 2010 criteria or Classification for Psoriatic Arthritis criteria, respectively [21,22].
2. LDA, defined as DAS28 ≥2.6 and <3.5 or DAPSA >4 and <16 [23,24].
3. Stable DMARD dosage and oral prednisone (or equivalent) at a dose ≤7.5 mg for ≥6 months prior to randomization.
4. Age ≥18 years.

#### Exclusion Criteria

The exclusion criteria for this study are as follows:

1. Vegan diet or lifestyle, or following a specific (medical) diet.
2. History of bariatric surgery.
3. Pregnant or nursing (lactating) women.
4. Underlying metabolic, hematologic, renal, hepatic, pulmonary, neurologic, endocrine, cardiac, infectious, or gastrointestinal conditions that, in the opinion of the investigator, place the patient at unacceptable risk for participation in a lifestyle intervention program.
5. Unable to understand, speak, and write in Dutch.

### Randomization and Blinding

Patients are randomized with a 1:1 allocation ratio into the intervention group (participating in the online, interactive lifestyle intervention program) or the control group (receiving general nutrition recommendations), using ALEA clinical software (ALEA Clinical B.V.). The allocation sequence was balanced for diagnosis (RA or PsA). Patients, treating physicians, RNs, and researchers will know to which group patients are randomized. The principal investigator will generate the allocation sequence and will assign participants to interventions using ALEA clinical software. RNs will enroll participants and do not have access to the randomization sequence. During statistical analysis, treatment allocation will remain concealed through the use of masked group labels.

### Interventions

#### Online, Interactive Lifestyle Intervention Program

The online, interactive lifestyle intervention program (Leef! met Reuma) consists of a 6-month, intensive part followed by an 18-month online aftercare period. The online program focuses on 4 pillars, namely nutrition, exercise, relaxation, and sleep. The general principles for each pillar are summarized in Table 1. All principles are explained in more detail during the online plenary sessions (the why and how behind them) through Zoom (Zoom Communications Inc), which can be accessed via a standard web browser. Participants are given tools to implement a healthier lifestyle and to increase their health skills. They are encouraged to implement those changes in ways that fit their daily life. The lifestyle intervention aims for lasting behavior changes (based on the I-Change model) [18]. The guidance team consists of a coach, a dietitian, a program coordinator, and experts on sleep, relaxation, and physical activity. The nutritional advice and diet recommended throughout the course are based on the Dutch Health Council guidelines and existing scientific literature. The diet that is prescribed is comparable to the Mediterranean diet, with an emphasis on unprocessed foods (especially vegetables) and 3 meals a day. Alcohol is excluded from the diet.

**Figure 1.**
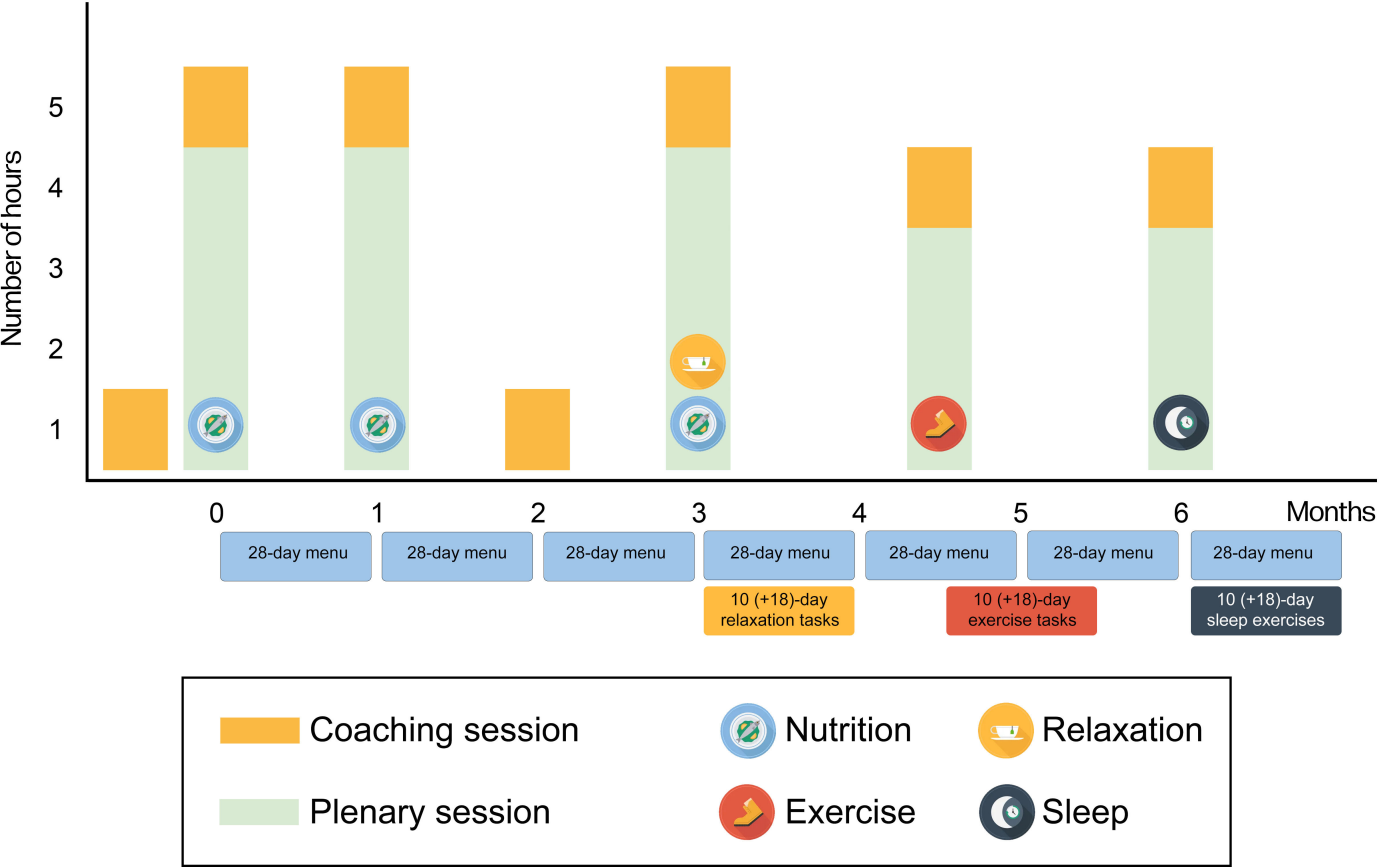
Schematic overview intervention (Leef! met Reuma).

Each month, a different pillar is highlighted. Throughout the month, participants are assigned 10 days of daily tasks focused on physical activity, relaxation, or sleep (with the option to extend this period to 28 d). Figure 1 provides a schematic overview of the intervention.

**Table 1.** General principles of Leef! met Reuma.

Pillar	Principles
Nutrition^a^	Eat unprocessed foods and vary in the foods you eat. Eat mostly plant-based. Pay attention to dietary quality and not solely energy content.
Physical activity	Strive to increase your heart rate with moderate-intense physical activity for at least 15‐30 min a day. Implement more physical activity during the day; all physical activity counts. Vary in the type of physical activity you do and find a type of physical activity that suits you and that you can enjoy.
Relaxation	Learn to notice when you are stressed and what causes you to be stressed. Find your personal way to implement relaxation. Control your breathing.
Sleep	Pay special attention to sleep quality and not only to the amount of sleep. Develop your own personal, effective sleep hygiene.

a Comparable to the Mediterranean diet.

There are 5 online plenary sessions lasting 3 to 4 hours for all participants (50 per group), and each session focuses on 1‐2 pillars. The online sessions start with a presentation with in-depth information about the specific pillar, and throughout the presentation, participants can ask questions. After that, the group is divided into several smaller online coaching groups. In these groups, the coach explains how to implement specific changes into participants’ lifestyles. During these 1-hour coaching sessions, the progress that participants have made will be discussed, but also obstacles, experiences, or situations that participants have encountered. Tips are given to help them stay motivated and keep the lifestyle behavioral changes they have made. One example is that participants will learn how to develop their own recipes according to the nutrition principles of the lifestyle program.

After the first 6 months, participants can participate in facultative sessions to stay motivated and foster the healthier habits they have learned during the intensive part of the program. These facultative sessions are similar to the coaching sessions but also include a recap of the knowledge gained during the first 6 months of the program. A facultative session is organized every 9 weeks. The total duration of these plenary, coaching, and facultative sessions is 16, 7, and 10 hours, respectively.

Throughout the entire program, participants can access the Voeding Leeft online platform to stimulate the implementation of healthier habits. At this platform, there are recipes for 30 days, and participants can also find more background information about all 4 pillars, ask questions (directed at the program coordinator, nutritionist, coach, or peers), chat with other participants to share their experiences and/or track their progress to ensure the implementation of a more lasting lifestyle behavior change.

Noteworthy is the fact that there will be no information exchange between the treating rheumatologist and the organization of the online, interactive lifestyle intervention program. However, for this trial, Voeding Leeft will record whether participants attend the plenary and coaching sessions and track their activity on the online platform.

#### General Nutrition Recommendations

The general nutrition recommendations are based on The Wheel of Five of the Dutch food-based dietary guidelines [25]. These recommendations are given in consultation with the RN. The general nutrition recommendation shows how much of what we eat should come from food groups to achieve a healthy, balanced diet. An overview of these recommendations is shown in Textbox 1. All patients at Erasmus MC’s rheumatology outpatient clinic receive this information as part of usual care. Therefore, this is chosen as a comparator for the intervention.

Textbox 1. **General nutrition recommendations for the control group, based on The Wheel of Five of the Dutch food-based dietary guidelines.**

- Eat at least 5 portions of a variety of fruit and vegetables a day.
  - They should make up over a third of the food we eat each day.
  - Choose from fresh, frozen, tinned, dried, or juiced.
  - Fruit juice and smoothies should be limited to no more than a combined total of 150 mL a day.
- Basic meals based on potatoes, bread, rice, pasta, or other starchy carbohydrates.
  - Starchy food should make up just over a third of the food we eat.
  - Choose higher-fiber whole-grain varieties, such as whole-wheat pasta and brown rice, or simply leave skins on potatoes.
- Have some dairy or dairy alternatives (ie, soya drinks or yogurts).
- Eat some beans, pulses, fish, eggs, meat, and other protein.
  - Aim for at least 2 portions of fish every week, 1 of which should be oily, such as salmon or mackerel.
- Choose unsaturated oils and spreads and eat them in small amounts.
- Eat foods high in fat, salt, and sugar less often and in small amounts.
- Drink plenty of fluids—the government recommends 6 to 8 cups or glasses a day.
  - Water, lower-fat milk, and lower-sugar or sugar-free drinks, including tea and coffee, all count.

### Strategies to Improve Adherence to Interventions

Patients have a personal responsibility for the implementation of the online lifestyle intervention program theory or general nutrition recommendations into their own lives. All food products are available at the local grocery store or butcher, and participants should buy them at their own expense. To measure adherence to the lifestyle program, a questionnaire is included to assess diet compliance. In addition, a sensitivity analysis will be performed on patients who have ≥5% weight loss after the intensive part of the lifestyle intervention program because we assume that this weight loss is associated with program adherence.

### Relevant Concomitant Care Permitted or Prohibited During the Trial

The use of DMARDs, oral prednisone (or equivalent) at a dose ≤7.5 mg as cointerventions are permitted if participants have received a stable dose for ≥6 months prior to randomization. Tapering, continuation, or intensification of treatment during the study is left to the discretion of the treating rheumatologist in consultation with the patient, using current treatment recommendations as a guideline. There are no specifically excluded concomitant medications.

Escape medication is defined as any new therapeutic intervention or a significant change to ongoing therapy made because a participant is experiencing worsening or exacerbation of their disease. Only the following prespecified escape medications are allowed:

1. A total of 80 mg triamcinolone acetonide or 120 mg methylprednisolone intramuscular (maximum of 1 per 3 mo).
2. Triamcinolone acetonide intra-articular ≤40 mg (maximum of 2 per 3 mo).
3. Nonsteroidal anti-inflammatory drugs.

### Criteria for Discontinuing or Modifying Allocated Interventions

Criteria for discontinuing are as follows:

1. Pregnancy.
2. Ineligibility (either arising during the trial or retrospectively having been overlooked at screening).
3. An adverse event (AE), which requires discontinuation of the lifestyle intervention or results in the inability to continue to comply with trial procedures.
4. Disease progression, which requires discontinuation of the lifestyle intervention or results in the inability to continue to comply with trial procedures.
5. Withdrawal of consent.
6. Loss to follow-up.

### AEs

The health risk of the intervention is expected to be low. AEs are defined as any undesirable experience occurring to a participant during the study, whether or not considered related to the intervention. All AEs reported spontaneously by the participant or observed by the investigator or research staff will be recorded. AEs of special interest during the study will be collected at each study visit through patient questionnaires and/or physician reports. Such AEs include the following:

1. Patient reported: infections (most common: [upper] airway), indigestion, diarrhea, and headache.
2. Physician reported: infections, abnormal blood counts, abnormal liver function tests, decrease in kidney function, and increased cholesterol levels.

A serious adverse event (SAE) is any untoward medical occurrence or effect that:

1. results in death; or
2. is life-threatening (at the time of the event); or
3. requires hospitalization or prolongation of existing inpatients’ hospitalization; or
4. results in persistent or significant disability or incapacity; or
5. is a congenital anomaly or birth defect; or
6. any other important medical event that did not result in any of the outcomes listed above due to medical or surgical intervention but could have been based upon appropriate judgment by the investigator.

An elective hospital admission will not be considered as an SAE. The investigator will report all SAEs that occur during the 2-year follow-up period to the sponsor without undue delay after obtaining knowledge of the events.

### Participant Timeline

**Figure 2.**
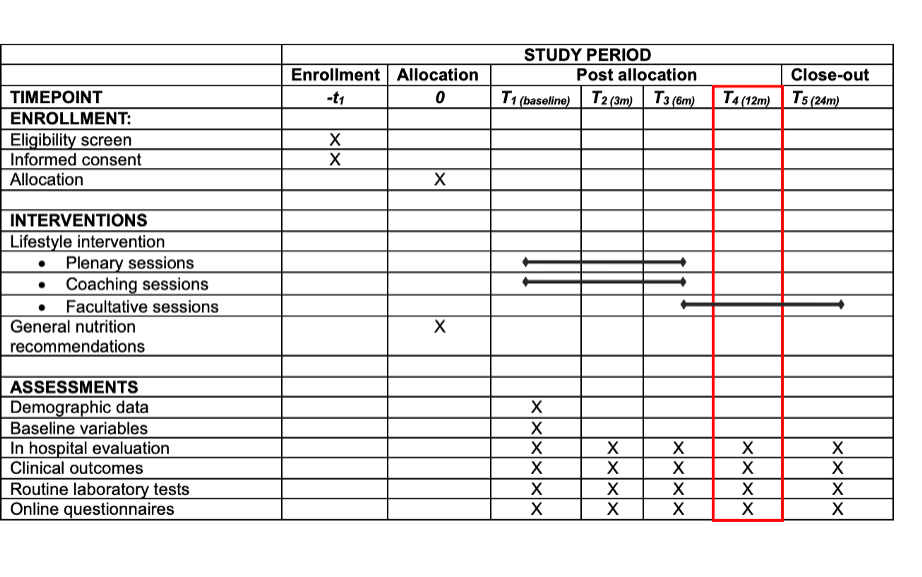
Participant timeline.

Patients will be assessed at baseline and after 3, 6, 12, and 24 months. At each visit, patients will fill out online questionnaires (~30 min) and are assessed by the RN, who calculates the DAS28 or DAPSA depending on the diagnosis. Study visits will take 30 minutes or less. The timeline and extensive data collection overview are shown in Figure 2 and Table 2. Data collection is the same for both the intervention group and the control group.

**Table 2.** Overview of data collection in intervention and control groups.

Data collection	T0	T3	T6	T12	T24
In-hospital evaluation					
Diagnosis: RA^a^ or PsA^b^	✓				
Check other inclusion and exclusion criteria	✓				
Informed consent	✓				
Demographic	✓				
History (fulfillment ACR^c^/and EULAR^d^ criteria for RA and PsA, disease duration, auto-antibody status, presence of erosions, DMARD^e^ history)	✓				
Comorbidity	✓				
Global assessment of disease activity by patient and RN^f^	✓	✓	✓	✓	✓
Adverse events (self-reported)		✓	✓	✓	✓
Hospital admission		✓	✓	✓	✓
Length, Weight, BMI, and waist circumference	✓	✓	✓	✓	✓
Physical examination by RN	✓	✓	✓	✓	✓
Online questionnaires					
General (smoking, alcohol, and drugs)	✓				
General health (VAS^g^)	✓	✓	✓	✓	✓
Pain (VAS)	✓	✓	✓	✓	✓
Morning stiffness (severity and duration; NRS^h^)	✓	✓	✓	✓	✓
Fatigue (VAS and FACIT-F^i^)	✓	✓	✓	✓	✓
HAQ^j^ (functional ability)	✓	✓	✓	✓	✓
EQ-5D-5L (quality of life)	✓	✓	✓	✓	✓
SF-36^k^ (quality of life)	✓			✓	✓
RAPID-3^l^	✓	✓	✓	✓	✓
Sleep (MOS-SS^m^)	✓	✓	✓	✓	✓
Perceived stress	✓	✓	✓	✓	✓
Physical activity	✓	✓	✓	✓	✓
Diet compliance	✓	✓	✓	✓	✓
WPAI^n^ (work and productivity)	✓	✓	✓	✓	✓
Medical consumption (MCQ^o^)	✓	✓	✓	✓	✓
Total time (min)	~34	~30	~30	~34	~34
Routine laboratory tests	✓	✓	✓	✓	✓

a RA: rheumatoid arthritis.

b PsA: psoriatic arthritis.

c ACR: American College of Rheumatology.

d EULAR: European Alliance of Associations for Rheumatology.

e DMARD: disease-modifying antirheumatic drug.

f RN: research nurse.

g VAS: Visual Analog Scale.

h NRS: Numeric Rating Scale.

i FACIT-F: Functional Assessment of Chronic Illness Therapy-Fatigue.

j HAQ: Health Assessment Questionnaire.

k SF-36: 36-item Short Form Health Survey.

l RAPID-3: Routine Assessment of Patient Index Data 3.

m MOS-SS: Medical Outcomes Study sleep scale.

n WPAI: Work Productivity and Activity Impairment.

o MCQ: Medical Consumption Questionnaire.

### Primary End Point

The primary objective is the proportional difference in DAS28 and DAPSA remission and/or successful tapering of DMARD treatment after 12 months of follow-up. Meeting either criterion is sufficient to qualify for the primary outcome.

Disease activity in patients with RA and PsA is measured with the DAS28 and DAPSA, respectively [23,24]. The DAS28 is a pooled index that involves the incorporation of a 28-joint count for tenderness (TJC28), a 28-joint count for swelling (SJC28), an erythrocyte sedimentation rate (ESR) and GH (measured with a Visual Analog Scale [VAS] 0‐100 mm) into a formula to obtain a numerical indicator of disease activity. The DAS28 formula is 0.56√(TJC28)+0.28√(SJC28)+0.70ln(ESR)+0.014(GH) [23]. The DAPSA is also a pooled index, which is made up of the following five domains: (1) a 68-joint count for tenderness, (2) a 66-joint count for swelling, (3) a CRP (expressed in mg/dL), (4) a patient’s assessment of the disease activity (VAS, 0‐10 cm), and (5) a patient’s assessment of pain severity (VAS, 0‐10 cm). The numerical values of the aforementioned 5 domains are summed to provide the DAPSA score [24]. The threshold values for DAS28 and DAPSA remission are <2.6 and ≤4, respectively [24,26]. The minimal clinically important differences (MCIDs) for the change in DAS28 and DAPSA are 1.2 and 7.2, respectively [27,28]. Tapering of treatment is left to the discretion of the treating rheumatologist in consultation with the patient, with current treatment recommendations as a guideline.

### Secondary End Points

Secondary end points include cost-effectiveness, health risk, and (self-reported) disease burden (refer to Table S1 in Multimedia Appendix 1). Cost-effectiveness will be evaluated through the ICER, which is the ratio of the difference in costs to incremental benefits between the online interactive lifestyle intervention program and general nutrition recommendations. For the cost-effectiveness analysis, we will calculate the quality-adjusted life years (QALYs) and total costs. QALYs express the impact of the disease on patients’ health over time. Living in perfect health corresponds to a QALY of 1 and a QALY of 0 reflects death [29]. QALYs are determined by calculating the area under the curve of the EQ-5D-5L [30]. Costs are divided into direct and indirect costs. We will analyze direct and indirect costs from a societal perspective. Direct costs are the costs of treatment and medical consumption, whereas indirect costs are costs due to loss of productivity [31]. Medication costs are calculated from doses reported in the patients’ case records, valued according to the Dutch National Health Care Institute [32]. Medical consumption, including duration of hospitalizations and admission diagnosis, are recorded every 3 months with the Institute for Medical Technology Assessment medical consumption questionnaire. We will use the Dutch average length of stay by diagnosis if the duration of hospitalization is unknown. Indirect costs include not fully functioning (presenteeism), sick leave (absenteeism), and reduction in work time [33]. Work Productivity and Activity Impairment (WPAI) outcomes are expressed as impairment percentages, with higher numbers indicating greater impairment and less productivity.

The health risk is assessed by measuring weight, BMI, and waist circumference. BMI is calculated by dividing the patient’s weight (kg) by the square of their height (m). A BMI ranging from 18.5 to <25 is classified as a healthy weight. A BMI between 25 and <30 is categorized as overweight, while a BMI ≥30 is considered obese. Conversely, a BMI <18.5 is classified as underweight [34]. Sex-specific thresholds for waist circumference are associated with an elevated risk of metabolic complications. In males, a waist circumference ≥94 cm is linked to an increased risk, whereas a measurement of ≥102 cm corresponds to a substantially increased risk. For females, these thresholds are ≥80 cm and ≥88 cm, respectively [34].

Disease burden is measured through the following PRO measurements: GH, pain, morning stiffness, fatigue, functional ability, and quality of life. GH, fatigue, and pain are measured with a VAS (0‐100 mm), whereby higher scores reflect greater severity. The MCIDs for the aforementioned outcomes are ≥10 [35-37]. Fatigue is also measured with the Functional Assessment of Chronic Illness Therapy-Fatigue questionnaire (FACIT-F) [38]. The FACIT-F consists of 13-items with a 7-day recall period. Items are scored on a 0‐4 response scale with anchors ranging from “Not at all” to “Very much so.” All items are summed to create a single fatigue score with a range from 0 to 52 and higher scores represent less fatigue [38]. The MCID is 15.9 [39]. Morning stiffness (severity and duration in min) is measured with a Numeric Rating Scale and is interpreted similarly to GH. The MCID for morning stiffness is ≥1 [40]. Functional ability is measured with the Health Assessment Questionnaire [41]. Higher Health Assessment Questionnaire scores indicate poorer function, and the MCID is 0.22 [41,42].

Quality of life is measured with the EQ-5D-5L (MCID ≥0.04) [43,44]. Higher scores represent a higher quality of life. We will also use the 36-item Short-Form Health Survey (SF-36) that assesses 8 domains on a scale of 0‐100, with higher scores indicating a better health status [45]. The SF-36 addresses the following domains: physical functioning, bodily pain, role limitations due to physical health problems and social or emotional problems, mental health, vitality, and GH perceptions. These domains are also combined into a physical and mental component score. The MCIDs for the physical component score and mental component score are 2.5 and 5, respectively [30,45,46].

Although the primary outcome will be assessed after 12 months of follow-up, we will follow all participants for 2 years to determine the long-term effect of the lifestyle intervention and whether the intervention has led to a permanent behavioral change.

### Exploratory End Points

Exploratory end points include self-reported disease activity (states), sleep, perceived stress, physical activity, and diet compliance (refer to Table S1 in Multimedia Appendix 1). Self-reported disease activity is measured with the Routine Assessment of Patient Index Data 3 (RAPID-3) [47]. Thresholds for remission and moderate-to-high disease activity are respectively <3.1 and ≥6.1 on a 0‐30 scale. The MCID for the RAPID-3 is 3.8 [48].

The quality of sleep is measured with the Medical Outcomes Study sleep scale (MOS-SS) [49]. The MOS-SS includes 12 items assessing sleep disturbance, sleep adequacy, somnolence, quantity of sleep, snoring, and awakening. A sleep problems index, grouping items from each of the former domains, is also available. The MCID for the sleep problem index is ≥6 [50].

Perceived stress is measured with the Perceived Stress Scale (PSS-10) [51]. The PSS-10 consists of 10 items, and items are scored on a 0‐4 response scale. Higher scores represent higher levels of stress. The MCID of perceived stress is ≥11 [52].

Physical activity is measured with the Dutch Physical Activity Guideline (Nederlandse Norm Gezond Bewegen [NNGB]) [53]. The NNGB measures whether an individual complies with the Dutch Guidelines for Physical Activity, and a higher score corresponds with better compliance.

Diet compliance is measured with a self-developed questionnaire consisting of 17 items based on the 17-item Mediterranean diet questionnaire [54]. Each dietary compliance item is scored as 1 if it aligns with the principles of the Mediterranean diet, and 0 otherwise. Total scores range from 0 to 17, with a score of 0 to 7 equating to a low dietary compliance, a score of 8 to 10 for an average degree of dietary compliance, and a score of 11 to 17 for a high degree of dietary compliance.

### Other Study Parameters

At baseline, we will also gather data on demographics and comorbidities. We will also perform a general physical examination. The demographic dataset will include age, sex, ethnicity, marital status, work status, education, smoking status, and alcohol consumption, as well as disease duration, autoantibody status, and presence of erosions. Furthermore, we will ask for the presence of the following comorbidities: diabetes, inflammatory bowel disease, cardiovascular diseases, urinary tract disorders, and malignancies. During the general physical examination, we will measure the height, weight, and waist circumference. Height and weight will be used to calculate the BMI.

### Sample Size

The HELIA is a superiority trial, powered to detect a 20% difference in DAS28 and DAPSA remission and/or successful tapering of DMARD treatment after 12 months of follow-up. Meeting either criterion is sufficient to qualify for the primary outcome. Murray et al [55] showed that after the first year of diagnosis, remission rates are up to 30%. Although tapering of treatment is still not standard of care, previous research has shown that tapering of treatment in patients with LDA is possible [56]. Based on these data, we assumed that 30% of the patients in the control group, general nutrition recommendations, will reach remission or be able to successfully taper their medication and that this will be 20% higher in patients who will follow the online, interactive lifestyle intervention program. Thus, to detect this 20% difference using a significance level of *α*=.05 (2-sided) and a power of 80%, 100 patients per group (~75 RA and ~25 PsA) are needed, also taking a 10% dropout ratio into account, which is based on previous trials conducted in the Erasmus MC [57,58].

### Statistical Analysis

#### Overview

Primary and secondary end points are analyzed according to an intention-to-treat principle, also known as full analyses. In an intention-to-treat analysis, all patients are analyzed in the groups to which they were randomized, regardless of whether they received or adhered to the allocated management approach.

Reporting of results will be in accordance with the CONSORT (Consolidated Standards of Reporting Trials) statement. Means (SD) will be presented for normally distributed data and medians (IQR) for nonnormally distributed data. Frequencies are presented as the number of patients (proportion).

We assume that missing data are (missing) at random. Therefore, missing data, in baseline variables as well as in the follow-up data, will be handled with multiple imputations by chained equations, with 40 imputations [59]. A Bonferroni correction will be applied if necessary to account for multiple testing by dividing the *P* value by the number of tests performed. All data analysis, including cleaning, transforming, and modeling, will be done in STATA18 (StataCorp LLC) or higher.

#### Primary End Point Analyses

The primary end point is the proportional difference in DAS28 and DAPSA remission and/or those who were able to taper their medication without having a disease flare after 12 months of follow-up between patients with IA who followed the lifestyle intervention program and those who received general nutrition recommendations [23,26]. Meeting either criterion is sufficient to qualify for the primary outcome. Logistic regression will be used to compare aforementioned differences at 12 months, in which intervention allocation, diagnosis (RA or PsA), biologic or targeted synthetic DMARD usage at randomization (yes or no), age (years), and sex are the covariates. If baseline imbalances in CRP, tender and swollen joint counts between both interventions occur, we will also correct for these imbalances.

#### Secondary End Points Analyses

The ICER will be used as an outcome for the cost-effectiveness analysis. The ICER is the ratio of the difference in total costs to incremental benefits (QALYs) between both management approaches. Total costs are divided into health care (direct) and productivity (indirect) costs, which will be analyzed from a societal perspective [31]. QALYs are calculated as the area under the curve of the EQ-5D-5L [30]. A probabilistic sensitivity analysis will be performed for the estimation of the ICER by bootstrapping with 1000 iterations using a Monte Carlo simulation. Results will be plotted in a cost-effectiveness plane, which will be used to estimate the 95% CI of the ICER. Additionally, a cost-acceptability curve will be extracted to show the probability of each intervention being cost-effective at different levels of willingness-to-pay thresholds in comparison with each other. Sensitivity analysis will be performed to assess the main drivers of cost-effectiveness.

Other secondary end points are health risk, weight, BMI and waist circumference, and PROs, including GH, pain, morning stiffness, fatigue, functional ability, and quality of life, at baseline, after 3 months, 6 months, 1 and 2 years of follow-up, and over time between both groups. A Student *t* test, chi-square test, or Wilcoxon rank-sum test will be used to compare the difference in aforementioned outcomes at fixed time points, when appropriate. Additionally, a mixed model with an unstructured covariance matrix will be used to compare outcomes over time. In all analyses, we will correct for our randomization minimization procedure. Moreover, the PRO difference will be weighed against the MCID.

#### Exploratory Study End Point Analyses

Self-reported disease activity (states), worker productivity, sleep, perceived stress, physical activity, and diet compliance are exploratory study parameters.

#### Other Study End Points Analyses

Other study end points are age, sex, disease duration, autoantibody status, presence of erosions, smoking status, and alcohol consumption, which are only compared at baseline. A Student *t* test, chi-square test, or Wilcoxon rank-sum test will be used to compare the difference in aforementioned outcomes, when appropriate.

#### Additional Analyses

Two additional analyses will be conducted to complement the primary analysis. First, we will conduct a complete case analysis that will include all participants who have never missed a study visit. Second, participants with ≥5% weight loss after the intensive part of the lifestyle intervention program will be compared with the complete case in the general nutrition group, because we assume that this weight loss will be associated with the greatest program compliance and thus its possible benefits.

### Data Collection and Management

#### Data Collection

Data will be collected electronically using GemsTracker (Generic Medical Survey Tracker; GemsTracker BV) and LimeSurvey (LimeSurvey GmbH). GemsTracker is a software package for (complex) distribution of questionnaires and forms during clinical research and quality registrations in health care. The software allows you to set up your own website for data collection. On a GemsTracker site, different users are able to submit, view, and send information. Within GemsTracker, it is possible to configure the rights and roles within the system, and it is possible to decline access to roles. The data entered into GemsTracker will be seen as the source and thus the electronic clinical report form (eCRF). The access and role within the eCRF—thus GemsTracker—will be assigned by the project coordinator. All rights and roles of those being able to log in to the eCRF are being logged.

LimeSurvey is an open-source software to securely create and use online questionnaires. All participants and a selection of designated and trained RNs will complete online questionnaires. Patients will not have access to the eCRF and thus to the data. Patients will receive an email including a link to complete the questionnaires. That link does not contain any personal data.

#### Data Storage

The software packages and all data are stored on a server hosted by the Sponsor, Erasmus MC. Data are stored for 15 years after the last visit of the last participant. Only a dedicated selection of programmers has access to the database on the server.

#### Data Confidentiality

All data will be handled confidentially. In addition, all staff will be trained before having access to the eCRF. All authors have access to the final dataset.

### Data Monitoring

The principal investigator ensures that monitoring procedures are performed before, during, and after the study. Prior to enrollment, a member of the study team will train staff on the procedures for obtaining informed consent, record keeping, and reporting of SAEs to the principal investigator. Monitoring includes inspection and reviewing eCRFs, participants’ source documents, and all other study documentation by a member of the study team in accordance with the study monitoring plan. All aspects of the study will be carefully monitored for compliance with applicable governmental laws and regulations concerning good clinical practice and the General Data Protection Regulation.

### Ethical Considerations

This study was conducted in accordance with the principles of the Declaration of Helsinki. The study protocol was approved by the local medical research ethics committee of the Erasmus MC in Rotterdam (MEC-2022‐0448). The protocol follows SPIRIT (Standard Protocol Items: Recommendations for Interventional Trials) guidelines (refer to SPIRIT checklist in Checklist 1). All study participants received full information about the study, and written informed consent was obtained from all individual study participants before inclusion. Through the consent form, study participants could also grant permission for secondary analyses in the future without additional consent. All study participants have the option to withdraw from the study at any time without providing a reason. The privacy and confidentiality of all study participants are guaranteed.

## Results

Due to administrative issues and time constraints, there has been a delay in registering in the ISCRTN registry. The study protocol was approved on December 6, 2022, by the ethics committee of the Erasmus MC. Patient recruitment began on March 21, 2023, while registration in the ISRCTN (International Standard Randomised Controlled Trial Number) registry was completed on June 13, 2023, which was fully based on the approved protocol by the ethics committee (ISRCTN21290281). Although prospective registration is generally recommended by the ICMJE (International Committee of Medical Journal Editors) guidelines, the authors acknowledge this deviation. They confirm that all study procedures and outcomes were predetermined prior to ethical approval and thus prior to data collection and that the outcomes and analysis plans have not been changed after registration. Recruitment was completed in May 2024. The last visit will take place in May 2026. Subsequently, the data will be analyzed, and the results are expected to be published in 2027.

## Discussion

### Principal Findings

The HELIA trial is a randomized controlled trial comparing the effectiveness of an online, interactive lifestyle intervention program and general nutrition recommendations in patients with IA with LDA. This study aims to investigate the effectiveness from a clinical, societal, and patients’ point of view. Considering the current developments in health care, such as the increasing number of chronically ill people, rising health care costs, and turnover of professionals, lifestyle is becoming increasingly important to prevent disease or reduce the burden of disease and indirectly reduce the pressure on health care.

The World Health Organization (WHO) promotes a healthy lifestyle for its benefits in preventing and slowing chronic diseases [60,61]. In rheumatic and musculoskeletal diseases (RMDs), an unhealthy lifestyle worsens the disease burden [62]. Accordingly, the EULAR recommends applying the WHO lifestyle guidance to RMD care as an essential complement to medical treatment. These recommendations include regular physical activity to improve pain, function, and quality of life, as well as maintaining a healthy weight and a balanced diet [63].

Although EULAR’s lifestyle recommendations are largely based on expert opinion, growing evidence supports the beneficial effects of lifestyle intervention programs. A recent pilot study on the effect of an online, interactive lifestyle intervention program (organized by Voeding leeft) in patients with RMDs showed positive results [20]. Reported health risks and disease burden decreased in 88 patients with IA, 105 patients with osteoarthritis, and 71 patients with fibromyalgia. Among patients with IA, significant improvements were observed during the intensive phase in weight (mean −4.2, SD 0.7  kg), BMI (mean −1.4, SD 0.3), and waist circumference (mean −3.9, SD 0.7  cm). After the aftercare period, these improvements were largely maintained (weight: mean −3.5, SD 1.0  kg; BMI: mean −1.1 , SD 0.3; waist circumference: mean −4.0, SD 1.0  cm). Disease burden, assessed through PRO measurements, also improved, with reductions in morning stiffness severity (mean −1 , SD 0) and sleep disturbance (mean −6.1, SD 1.4) during the intensive phase. Over 24 months, significant improvements persisted for pain (mean −8, SD 3), morning stiffness (mean −1, SD 0), and sleep disturbance (mean −5.9, SD 1.8). However, this study had notable limitations, namely, no data on disease activity in patients with IA, no control group, and no assessment of social impact.

Another lifestyle intervention study, called “Plant for Joints,” also showed promising results, including reduced disease activity in RA, improved stiffness, pain, and function in osteoarthritis, and better metabolic status in both groups [16,17]. Yet, similar to the pilot study, data on disease burden and societal impact were limited. Given the small number of lifestyle intervention studies in rheumatology and generally low level of evidence, we set up the HELIA trial and chose our outcomes based on the aforementioned knowledge gaps.

With the HELIA trial, we hope to prove that lifestyle interventions can be integrated into standard care for patients with IA alongside DMARD treatment. The program’s strength lies in its focus on multiple lifestyle domains—nutrition, exercise, relaxation, and sleep—with the aim of achieving lasting behavioral change (based on the I-Change model) [18]. The study design also includes several strong features, such as a randomization procedure and an extensive follow-up period after the lifestyle intervention. The online, interactive lifestyle intervention, organized by Voeding Leeft, has also shown success in other patient groups, including the Reverse Diabetes 2 program [19]. We hope that the results of this study will encourage policymakers and insurers to recognize lifestyle interventions as reimbursable components of IA care.

### Strengths and Limitations of This Study

The strengths and limitations of this study are as follows:

1. The online, interactive lifestyle intervention, organized by Voeding Leeft, has already proved successful with other patient groups.
2. The intervention addresses multiple lifestyle factors: diet, exercise, relaxation, and sleep, with the aim of achieving a durable behavioral change.
3. The study design contains several strong features, including the long follow-up period and the inclusion of both patients with RA and PsA, which is a good representation of patients with IA.
4. The intervention is evaluated from a clinical, societal, and patients’ point of view.

## Supplementary material

10.2196/83749 Multimedia Appendix 1 An overview of trial outcomes and the informed consent form.

10.2196/83749 Checklist 1 SPIRIT checklist.

## Abbreviations

AE: adverse event
CONSORT: Consolidated Standards of Reporting Trials
CRP: C-reactive protein
DAPSA: Disease Activity in Psoriatic Arthritis
DAS28: Disease Activity Score
DMARD: disease-modifying antirheumatic drug
eCRF: electronic clinical report form
ESR: erythrocyte sedimentation rate
EULAR: European Alliance of Associations for Rheumatology
FACIT-F: Functional Assessment of Chronic Illness Therapy-Fatigue questionnaire
GH: general health
HELIA: Healthy Living in Inflammatory Arthritis
IA: inflammatory arthritis
ICER: incremental cost-effectiveness ratio
ICMJE: International Committee of Medical Journal Editors
ISRCTN: International Standard Randomised Controlled Trial Number
LDA: low disease activity
MCID: minimal clinically important difference
MOS-SS: Medical Outcomes Study sleep scale
PRO: patient-reported outcome
PsA: psoriatic arthritis
PSS-10: 10-item Perceived Stress Scale
QALY: quality-adjusted life year
RA: rheumatoid arthritis
RAPID-3: Routine Assessment of Patient Index Data 3
RMD: rheumatic and musculoskeletal disease
RN: research nurse
SAE: serious adverse event
SF-36: 36-item Short Form Health Survey
SJC28: 28-joint count for swelling
SPIRIT: Standard Protocol Items: Recommendations for Interventional Trials
TJC28: 28-joint count for tenderness
VAS: Visual Analog Scale
WHO: World Health Organization
WPAI: Work Productivity and Activity Impairment
