# Supplementary material for: Effect of an Online, Interactive Lifestyle Intervention Program on 12-Month Disease Management Outcomes: Protocol for The Healthy Living in Inflammatory Arthritis (HELIA) Randomized Controlled Trial
Source: JMIR Res Protoc. 2026 May 25;15:e83749. doi: 10.2196/83749 (PMC13200808; doi:10.2196/83749)
Supplement: Multimedia Appendix 1 [file resprot-v15-e83749-s001.docx]

**Table S1.** Overview of outcomes.

| *Assessment of clinical outcomes:*   - Disease activity measured with the DAS28 in RA and DAPSA in PsA (and whether or not remission is achieved). |
| --- |
| *Assessment of societal outcomes:*   - Quality of life, measured with the Dutch EuroQol questionnaire with 5 dimensions (EQ-5D-5L). - Worker productivity, measured with the Work Productivity and Activity Impairment (WPAI). - Medical consumption, measured with the iMTA Medical Consumption Questionnaire. |
| *Assessment of health risk*   - Weight (in kilograms). - Body Mass Index (BMI,in kg/m^2^). - Waist circumference (in centimetres). |
| *Assessment of patient reported outcomes (PROs):*   - General health, measured with a Visual Analogue Scale (VAS). - Pain, measured with a VAS. - Morning stiffness (severity and duration), measured with a Numeric Rating Scale (NRS). - Fatigue, measured with a VAS and the Functional Assessment of Chronic Illness Therapy – Fatigue (FACIT-F). - Functional ability, measured with the Health Assessment Questionnaire (HAQ). - Quality of life, measured with the EQ-5D-5L and 36-item Short Form Health Survey. |
| *Exploratory outcomes:*   - Self-reported disease activity measured with the Routine Assessment of Patient Index Data 3 (RAPID-3). - Quality of sleep, measured with the Medical Outcomes Study sleep scale (MOS-ss). - Perceived stress, measured with the Perceived Stress Scale (PSS-10). - Physical activity, measured with the Dutch Standard for Healthy Exercise questionnaire (NNGB). - Diet compliance, measured with a self-developed questionnaire consisting of 17 items based on the 17-item MedDiet questionnaire. |

A healthy lifestyle in inflammatory arthritis - May 2022

**Informed consent form (Translated from Dutch)**

Related to a healthy lifestyle in inflammatory arthritis research (HELIA trial).

- I read the information letter. I was also able to ask questions. My questions were answered well enough. I had enough time to decide whether to participate.
- I know that participation is voluntary. I also know that I can decide at any time not to participate in the study after all. Or to stop. I don't have to say why I want to stop.
- I give the researcher permission to inform my GP that I am participating in this study.
- I give the researcher permission to give my GP or specialist information about unexpected findings from the study that are relevant to my health.
- I consent to the collection and use of my data. The researchers do this only to answer the research question of this study.
- I know that for monitoring the study, some people can see all my data. These people are listed in this information letter. I give these people permission to see my data for this audit.
- I know I should preferably not get pregnant during the study.

Please tick yes or no in the table below?

| I consent to the storage of my data to be used for other research, as mentioned in the information letter. | Yes ☐ No☐ |
| --- | --- |
| I consent to being asked if I want to participate in a follow-up study if needed after this study. | Yes ☐ No☐ |

I want to participate in this study.

My name is (test subject): ………………………………………………………..

Signature: ………………………………………………………………………….. Date : __ / __ / __
--------------------------------------------------------------------------------------------------------------------------------------------

I declare that I have fully informed this subject of the aforementioned research.

If information becomes known during the study that may affect the consent of the subject, I will let the subject know in good time.

Investigator's name (or its representative):………………………………………

Signature:…………………………………………………………………………… Date: __ / __ / __
-----------------------------------------------------------------------------------------------------------------

The subject will receive a full information letter, together with a signed version of the consent form.
